# Evolution of the BRICS Agricultural Product Trade Network: Evidence from Social Network and QAP Analyses

**DOI:** 10.3390/foods15213897

**Published:** 2026-10-30

**Authors:** Xin Dai, Rui Han, Xiaoyu Jiang, Zhong Li, Ye Liu

**Affiliations:** Agricultural Information Institute, Chinese Academy of Agricultural Sciences, Beijing 100081, China; daixin@caas.cn (X.D.); jiangxiaoyu@caas.cn (X.J.); lizhong@caas.cn (Z.L.); liuye01@caas.cn (Y.L.)

**Keywords:** BRICS, agricultural product trade network, social network analysis, quadratic assignment procedure, institutional distance

## Abstract

Amid mounting global food risks, the expansion of BRICS—an important multilateral platform for emerging markets and developing economies—has reshaped agricultural trade relations within BRICS, carrying profound implications for food security and sustainable agricultural development. This study aims to analyze the evolution of BRICS agricultural product trade networks to deliver empirical insights for coordinated agricultural governance among BRICS members. We adopt a social network analysis (SNA) and the quadratic assignment procedure (QAP) to examine BRICS agricultural product trade network characteristics and identify its driving factors. The results reveal that agricultural product trade within BRICS expanded markedly after BRICS enlargement, accompanied by declines in network density and reciprocity. Furthermore, nodes in this agricultural trade network show growing functional differentiation: Brazil serves as the core supply hub, China the primary demand hub, and Russia a vital connecting node. Community analysis indicates that the network has not evolved into closed geographic blocs; instead, cross-regional linkages give rise to a polycentric hierarchical structure. QAP estimates show that agricultural supply capacity dominated network configurations before enlargement, while food-security pressure and institutional distance exerted more pronounced impacts afterward. These findings provide empirical evidence and policy references for BRICS members to deepen agricultural trade cooperation, stabilize cross-border agricultural supply chains, and collectively safeguard food security.

## 1. Introduction

As climate change, geopolitical conflicts and trade protectionism increase uncertainty in cross-border agricultural supplies, food security has become a central concern in global agricultural governance, extending beyond domestic production capacity to encompass the stability and resilience of transnational supply systems [1,2]. A country’s access to stable, diversified and affordable agricultural products depends not only on its domestic resource endowments, production capacity and purchasing power but also on its position in international agricultural product trade networks [3]. For emerging markets and developing economies, strengthening agricultural trade cooperation is an important means of securing agricultural supplies. BRICS serves as an important platform for cooperation among these economies and has made agricultural development and food security key priorities on its cooperation agenda [4]. Examining the evolution of the BRICS agricultural product trade network and its driving factors is therefore important for deepening agricultural trade cooperation among members, stabilizing cross-border agricultural supplies and safeguarding food security.

BRICS members are located across Asia, Europe, Africa and Latin America, account for approximately 48.5% of the world’s population, and differ substantially in resource endowments, agricultural production conditions, market demand and institutional environments. Unlike the Association of Southeast Asian Nations (ASEAN), the Regional Comprehensive Economic Partnership (RCEP) or the African Continental Free Trade Area (AfCFTA), which are largely supported by geographical proximity or regional institutional arrangements, BRICS does not rely on contiguous geographical space. Agricultural product trade among BRICS countries is therefore not conventional intra-regional trade. Its agricultural product trade network formation reflects supply–demand gaps and complementary potential among members, while also being shaped by institutional coordination, standards recognition and trade facilitation [5].

Existing studies suggest that agricultural product trade should be understood not only through bilateral flows but also through the linkage effects embedded in trade networks [6]. Against this background, this study focuses on BRICS agricultural product trade and constructs-directed weighted trade networks for five benchmark years: 2007, 2011, 2015, 2020 and 2024. These years cover important stages in the development of BRICS cooperation, from the early formation of the mechanism to the 2024 enlargement. The study addresses two questions. First, how have the overall structure, node roles and community structure of the BRICS agricultural product trade network evolved? Second, how do agricultural supply capacity, import demand pressure, supply–demand complementarity and trade resistance affect agricultural product trade linkages under a cross-regional institutional cooperation framework? By answering these questions, the study seeks to clarify both the evolutionary features and the formation logic of agricultural product trade relations within BRICS.

This study contributes to the literature in three ways. First, it takes BRICS as a cross-regional and institution-based cooperation platform, thereby extending agricultural product trade network research beyond geographically proximate regional arrangements. Second, it combines social network analysis with QAP methods by treating countries as nodes and bilateral agricultural product export values as directed weighted edges. This approach characterizes the network structure while accounting for the relational dependence of dyadic trade data. Third, it links the extended gravity-model logic of trade attraction, trade matching and trade resistance with the institutional features of BRICS cooperation.

## 2. Literature Review

BRICS cooperation developed with the rising influence of large emerging market economies in global governance. Brazil, Russia, India and China formed the initial BRIC dialog, and South Africa joined in 2011 [4,7]. Over time, the BRICS cooperation expanded from macroeconomic coordination to trade, agriculture, food security and sustainable development. Agricultural cooperation has been advanced through ministerial meetings and related policy platforms, while the 2024 enlargement further broadened the range of agricultural resources, market demand and institutional conditions represented in the group. Existing research related to this study can be organized into three strands: BRICS agriculture, agricultural trade networks and factors associated with agricultural trade.

### 2.1. Research on BRICS Agriculture

Research on BRICS agriculture has gradually moved from broad qualitative discussions toward more specific quantitative analyses. Earlier studies mainly examined BRICS agricultural cooperation from the perspectives of emerging economies, South–South cooperation and international economic governance, emphasizing its relevance to changes in the global economic order and food security [7]. More recent studies have focused on agricultural production capacity, resource use, technological innovation and sustainable agriculture [8,9,10]. Quantitative evidence suggests that agricultural technology, resource-use efficiency and production organization affect the agricultural output capacity of BRICS members [11,12,13], thereby shaping the basic conditions under which they participate in agricultural product trade. Studies on BRICS agricultural product trade have mainly examined competitiveness, complementarity, trade facilitation, trade efficiency and trade potential [14,15,16]. Methodologically, this literature often uses gravity models, stochastic frontier models, trade indices and network measures to assess how infrastructure, trade facilitation and the business environment influence agricultural product trade [17,18,19,20].

### 2.2. Research on Agricultural Product Trade Networks

From a network perspective, trade relations are not only characterized by scale but also by direction, intensity and position. Indicators such as network density, reciprocity, centrality, community structure and core–periphery structure can be used to identify connectivity, node roles and structural differentiation within trade networks [21,22,23,24], which complement conventional trade indicators.

Research on global agricultural product trade networks has shifted from the analysis of trade scale and bilateral relations toward network structure, node functions and risk transmission. Wang et al. examined global agricultural product trade networks from 1986 to 2018 and showed that agricultural product trade communities evolved from a single-dominant pattern toward a more multipolar structure, with differences in network stability affecting the resilience of the global food supply system [3]. Ji et al., focusing on rice, maize, wheat and soybean trade networks, found that the global food trade network displayed scale-free and small-world properties: a small number of major producers connect with many trading partners, emerging economies are more important, and rising import dependence in some economies increases network vulnerability [25]. Hedlund et al. incorporated climate change into the analysis of global food trade networks and showed that climate shocks can be transmitted across borders through international trade ties, reshaping food supply risks across countries [26]. Laber et al. constructed an international multilayer food production network and found that local agricultural production shocks could generate both direct impacts through trade linkages and indirect impacts through food-production conversion pathways [27].

The studies of regional agricultural product trade networks highlight the role of institutional arrangements and cooperation frameworks in shaping network structures. Evidence from China–ASEAN, RCEP, Africa and the Belt and Road Initiative shows that regional cooperation can alter trade connectivity, core nodes and community relations among members [28,29,30,31]. However, most of these studies are based on regional economic cooperation or geopolitical proximity, and their samples therefore have strong spatial attributes.

### 2.3. Research on Driving Factors of Agricultural Product Trade

Research on the determinants of agricultural product trade commonly begins with the gravity model. The traditional gravity model emphasizes economic scale and distance costs, while extended versions incorporate tariffs, common language, common borders, institutional quality and other factors [32,33,34,35,36]. In agricultural product trade, product characteristics and regulatory requirements create additional constraints. Production capacity, demand pressure, standards differences and institutional environments have therefore become important explanatory variables [37].

Supply-side and demand-side factors are central to agricultural product trade research [38]. On the supply side, existing studies emphasize the role of technological progress, resource-use efficiency and productivity growth in shaping agricultural exports [39]. Aragie et al. further show that export-oriented agricultural policies may involve trade-offs between export growth and food accessibility when countries face international market volatility or declining domestic productivity [40]. On the demand side, scholars examine how consumption structures, resource constraints and environmental conditions influence agricultural imports [41]. Supply–demand complementarity mainly refers to the transactional supply–demand alignment between exporters and importers at the aggregate agricultural product trade level, which is also important for explaining agricultural trade linkages. Liu et al., using export similarity and trade complementarity indices, show that agricultural product trade among Belt and Road economies involves both competition and complementarity, with matched and mismatched relations coexisting [42].

### 2.4. Review Summary and Analytical Framework

Overall, existing studies provide an important basis for understanding agricultural product trade relations, but three gaps remain. First, research on BRICS agricultural product trade has focused mainly on bilateral trade relations and trade potential, while paying less attention to the overall network structure among members. Second, agricultural product trade network studies have concentrated on global systems or geographically proximate regional mechanisms, whereas cross-regional cooperation platforms such as BRICS remain underexplored. Third, although the set of explanatory variables has expanded, the relational dependence of the trade network data has not always been adequately addressed. These gaps are particularly relevant for understanding how cross-regional agricultural cooperation contributes to food-system resilience under changing global trade conditions.

**Figure 1:**
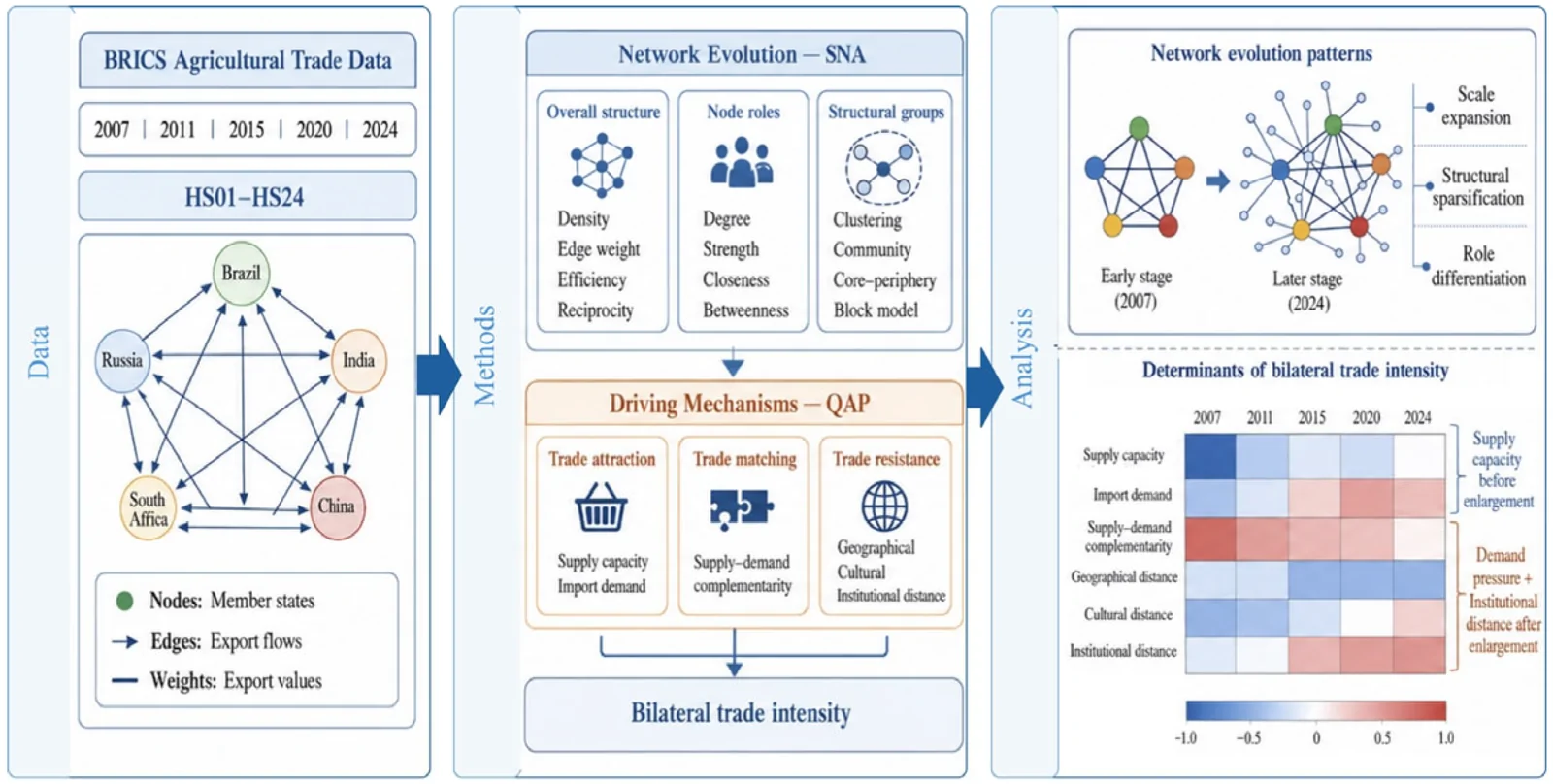
Research framework.

Building on these studies, this paper organizes the analysis around four dimensions: trade attraction, trade matching, trade resistance and network embeddedness. Trade attraction includes agricultural supply capacity and import demand pressure; trade matching is reflected in supply–demand complementarity; trade resistance includes geographical, cultural and institutional distance; and network embeddedness refers to the relational position of member states within the trade network (Figure 1). This framework connects the extended gravity model with social network analysis and provides an integrated perspective for explaining how agricultural product trade linkages are formed, how network positions differ across members, and how institutional and supply–demand conditions affect the resilience of cross-regional agri-food cooperation.

## 3. Methods and Data

Building on the analytical framework above, this study integrates a social network analysis (SNA) with the quadratic assignment procedure (QAP). A directed and weighted network is constructed in which BRICS members are represented as nodes, bilateral agricultural product exports as directed edges, and export values as tie weights. SNA is used to analyze changes in the overall network structure, the positions and roles of individual members, and the formation of structural groups. The analysis then draws on the extended gravity model to examine how agricultural supply capacity, import demand pressure, supply–demand complementarity, geographical distance, cultural distance, and institutional distance are associated with bilateral trade relations. And QAP correlation and regression analyses are employed to accommodate the relational dependence among country pairs and to evaluate these associations through matrix permutations. The following subsections present the construction of the trade network, the measurement of network characteristics, the specification of the QAP model and variables, and the procedures used for data processing and empirical implementation.

### 3.1. Construction of the BRICS Agricultural Product Trade Network

Each country’s trade position depends not only on its own trade volume but also on how it is embedded in the overall structure of trade relations [25]. To examine the structural evolution of agricultural product trade relations among BRICS members, this study constructs a directed weighted trade network for each benchmark year. In this study, agricultural products are defined according to Chapters 01–24 of the Harmonized Commodity Description and Coding System (HS01–HS24), covering live animals, animal and plant products, food preparations, beverages, tobacco and related agricultural commodities (see Appendix A, Table A1 for the full HS01–HS24 chapter). Bilateral trade data for HS01–HS24 products are obtained from the UN Comtrade Database and aggregated at the country-pair level. Compared with aggregate trade indicators, this directed weighted network captures the direction, intensity and asymmetry of agricultural trade flows among members.

For year *t*, the BRICS agricultural product trade network is defined as

*G^t^* = (*V^t^*, *E^t^*, *W^t^*) (1)

where *V^t^* denotes the set of BRICS members included in year *t*, *E^t^* denotes the set of directed agricultural product trade ties among members, and $W^{t}=\left[w_{\mathrm{ij}}^{t}\right]$ denotes the weighted trade matrix. Each country is treated as a node. If country *i* exports agricultural products to country *j*, a directed edge from *i* to *j* is established. The weight of this edge is the export value from country *i* to country *j*. Thus, the direction of the edge represents the exporter–importer relationship, while the weight represents the intensity of the trade flow.

The weighted adjacency matrix is expressed as

(2) $$W^{t}=\left[\begin{array}{cccc} 0 & w_{12}^{t} & \ldots & w_{1n}^{t}\\ w_{21}^{t} & 0 & \ldots & w_{2n}^{t}\\ \vdots & \vdots & \ddots & \vdots \\ w_{n1}^{t} & w_{n2}^{t} & \ldots & 0 \end{array}\right]$$

where $w_{\mathrm{ij}}^{t}$ represents the agricultural product export value from country *i* to country *j* in year *t*. The diagonal elements are set to zero because self-trade is not considered. Since exports from *i* to *j* and exports from *j* to *i* are two different trade relations, the matrix is generally asymmetric.

### 3.2. Overall Indicators of the Agricultural Product Trade Network

To characterize the overall structure of the BRICS agricultural product trade network, this study measures network-level indicators including network density, average edge weight, network efficiency, average path length and reciprocity. These indicators are calculated based on the directed weighted trade network. Together, they describe the connectivity, trade intensity, reachability and mutuality of the trade network, thereby providing a basis for assessing whether the expansion of BRICS agricultural product trade has been accompanied by deeper network integration and more balanced reciprocal relations among member states.

#### 3.2.1. Network Density

Network density measures the degree to which potential trade relations among BRICS members are realized. In a directed network with *n* nodes, the maximum number of possible directed edges is *n*(*n* − 1). Let *m^t^* denote the number of observed directed trade ties in year *t*. The network density is defined as

(3) $$D^{t}=\frac{m^{t}}{n^{t}(n^{t}-1)}$$

where *D^t^* ranges from 0 to 1. A higher density indicates that a larger proportion of potential agricultural product trade relations among BRICS members have been realized, implying stronger overall connectivity. A lower density suggests that many potential bilateral trade relations remain weak.

#### 3.2.2. Average Edge Weight

The average edge weight reflects the average trade intensity of existing trade ties. It is calculated as the total agricultural product trade value within the network divided by the number of observed directed edges:

(4) $$\bar{W^{t}}=\frac{\sum_{i}\sum_{j}w_{\mathrm{ij}}^{t}}{m^{t}},i\neq j,w_{\mathrm{ij}}^{t}>0$$

where $w_{\mathrm{ij}}^{t}$ is the export value from country *i* to country *j*, and *m^t^* is the number of existing directed trade ties. This indicator captures the average scale of trade flows among connected country pairs. A higher average edge weight indicates that existing trade relations are associated with larger trade volumes.

#### 3.2.3. Network Efficiency

Network efficiency measures how efficiently countries in the network can reach one another through trade paths. It is based on the inverse of the shortest path length between country pairs. Let $d_{\mathrm{ij}}^{t}$ denote the shortest path length from country *i* to country *j* in year *t*. The global network efficiency is defined as

(5) $$E^{t}=\frac{1}{n^{t}(n^{t}-1)}\sum_{i\neq j}\frac{1}{d_{\mathrm{ij}}^{t}}$$

where if country *j* is not reachable from country *i*, the corresponding term is treated as zero. A higher value of network efficiency indicates stronger reachability and a more efficient trade network structure. In the context of agricultural product trade, higher efficiency suggests that trade relations are more easily connected across members.

#### 3.2.4. Average Path Length

Average path length reflects the average number of steps required for one country to reach another through directed trade paths. It is calculated as

(6) $$L^{t}=\frac{1}{N_{r}^{t}}\sum_{i\neq j}d_{\mathrm{ij}}^{t}$$

where $d_{\mathrm{ij}}^{t}$ is the shortest path length from country *i* to country *j*, and $N_{r}^{t}$ is the number of reachable ordered country pairs. A smaller average path length indicates that countries are more closely connected and that trade relations can be reached through fewer intermediate steps. A larger value suggests a more dispersed or less integrated network structure.

#### 3.2.5. Reciprocity

Reciprocity measures the extent to which trade relations between country pairs are mutual. In the BRICS agricultural product trade network, a pair of countries is reciprocal if country *i* exports to country *j* and country *j* also exports to country *i*. Let *M^t^* denote the number of country pairs with mutual directed trade ties. Reciprocity is calculated as

(7) $$R^{t}=\frac{M^{t}}{n^{t}(n^{t}-1)/2}$$

where $n^{t}\left(n^{t}-1\right)/2$ is the total number of unordered country pairs. The value of *R^t^* ranges from 0 to 1. A higher value indicates that trade relations are more mutually connected, while a lower value indicates stronger asymmetry in agricultural product trade relations.

### 3.3. Node-Level Indicators of the Agricultural Product Trade Network

To identify the position and functional role of each member within the BRICS agricultural product trade network, this study calculates node-level indicators including out-degree, in-degree, out-strength, in-strength, closeness centrality and betweenness centrality. Degree indicators describe the number of trade partners, strength indicators describe the scale of trade flows, and centrality indicators capture accessibility and intermediary roles in the network.

#### 3.3.1. Out-Degree

Out-degree measures the number of countries to which a given country exports agricultural products. For country *i* in year *t*, out-degree is defined as

(8) $$OD_{i}^{t}=\sum_{j=1,j\neq i}^{n}a_{\mathrm{ij}}^{t}$$

where $a_{\mathrm{ij}}^{t}=1$ if country *i* exports agricultural products to country *j*, and $a_{\mathrm{ij}}^{t}=0$ otherwise. A higher out-degree indicates that the country has a wider range of export destinations within the BRICS agricultural product trade network.

#### 3.3.2. In-Degree

In-degree measures the number of countries from which a given country imports agricultural products. For country *i* in year *t*, in-degree is defined as

(9) $$ID_{i}^{t}=\sum_{j=1,j\neq i}^{n}a_{\mathrm{ji}}^{t}$$

A higher in-degree indicates that the country imports agricultural products from a larger number of BRICS partners, suggesting a greater diversification of import sources within the network.

#### 3.3.3. Out-Strength

Out-strength measures the total value of agricultural products exported by a country to other BRICS members. It is calculated as

(10) $$OS_{i}^{t}=\sum_{j=1,j\neq i}^{n}w_{\mathrm{ij}}^{t}$$

where $w_{\mathrm{ij}}^{t}$ represents the export value from country *i* to country *j*, A higher out-strength indicates that the country plays a stronger supplier role in the BRICS agricultural product trade network.

#### 3.3.4. In-Strength

In-strength measures the total value of agricultural products imported by a country from other BRICS members. It is calculated as

(11) $$IS_{i}^{t}=\sum_{j=1,j\neq i}^{n}w_{\mathrm{ji}}^{t}$$

A higher in-strength indicates that the country is a major demand-side node in the network and has stronger agricultural product import links with other BRICS members.

#### 3.3.5. Closeness Centrality

Closeness centrality measures the accessibility of a country within the agricultural product trade network. In a directed trade network, accessibility depends on the direction of trade flows. Therefore, this study calculates closeness centrality separately for export direction and import direction. Export closeness centrality reflects how efficiently a country can reach other members through outgoing trade paths, while import closeness centrality reflects how efficiently a country can be reached through incoming trade paths. Because the directed trade network may not be fully connected, this study adopts the reachability-adjusted closeness centrality. For country *i* in year *t*, closeness centrality is defined as

(12) $$CC_{i}^{t}=\frac{r_{i}^{t}}{n^{t}-1}\times \frac{r_{i}^{t}}{\sum_{j}d_{\mathrm{ij}}^{t}}$$

where $d_{\mathrm{ij}}^{t}$ is the shortest path length from country *i* to country *j*, $r_{i}^{t}$ is the number of countries reachable from country *i*, and *n^t^* is the number of countries in the network. The first term adjusts for the proportion of reachable countries, while the second term captures the inverse of the total shortest-path distance to reachable countries. A higher value indicates that the country is more accessible within the network and can connect with other members through fewer intermediate trade paths.

#### 3.3.6. Betweenness Centrality

Betweenness centrality measures the extent to which a country acts as an intermediary or bridge in the agricultural product trade network. A country with high betweenness centrality lies on a larger proportion of the shortest directed trade paths between other countries and may therefore play an important connecting role in the network structure.

Following the classic shortest-path-based definition of Freeman and its standard implementation for directed networks, the betweenness centrality for country *i* in year *t* is calculated as

(13) $$BC_{i}^{t}=\frac{\sum_{s\neq i\neq r}\frac{\sigma_{\mathrm{sr}}^{t}(i)}{\sigma_{\mathrm{sr}}^{t}}}{\left(n^{t}-1)(n^{t}-2\right)}$$

where $\sigma_{\mathrm{sr}}^{t}$ is the total number of shortest paths from country *s* to country *r*, and $\sigma_{\mathrm{sr}}^{t}(i)$ is the number of those shortest paths that pass through country *i*. The denominator (*n^t^* − 1)(*n^t^* − 2) standardizes the measure for a directed network, making the values comparable across networks with different numbers of nodes. A higher betweenness centrality indicates that a country has a stronger bridging or intermediary function.

### 3.4. Community and Structural Indicators of the Agricultural Product Trade Network

To further examine whether the BRICS agricultural product trade network forms closely connected subgroups or differentiated structural roles, this study analyzes the clustering coefficient, modularity, core–periphery patterns and a role-based block model. These indicators complement network-level and node-level measures by revealing local agglomeration, community division, hierarchical structure and trade flows among different types of countries.

#### 3.4.1. Clustering Coefficient

The clustering coefficient measures the tendency of countries and their trade partners to form locally connected groups. In this study, the clustering coefficient is calculated by first considering the connection structure derived from the trade network and then measuring the degree of local closure among neighboring countries. For country *i*, the local clustering coefficient is defined as

(14) $$C_{i}^{t}=\frac{2e_{i}^{t}}{k_{i}^{t}(k_{i}^{t}-1)}$$

where $k_{i}^{t}$ is the number of neighboring countries connected to country *i*, and $e_{i}^{t}$ is the number of actual connections among these neighboring countries. The overall clustering coefficient is the average of local clustering coefficients across all countries:

(15) $$C^{t}=\frac{1}{n^{t}}\sum_{i=1}^{n^{t}}C_{i}^{t}$$

A higher clustering coefficient indicates that countries tend to form closely connected trade groups, suggesting stronger local agglomeration within the agricultural product trade network; a lower value suggests that trade relations are more dispersed and less clustered.

#### 3.4.2. Modularity

Modularity is used to identify whether the network contains communities, namely groups of countries with stronger internal trade connections than external connections. Because community detection focuses on the strength of mutual trade relations, the directed weighted trade matrix is transformed into an undirected weighted matrix:

(16) $$W_{\mathrm{ij}}^{u,t}=w_{\mathrm{ij}}^{t}+w_{\mathrm{ji}}^{t}$$

where $W_{\mathrm{ij}}^{u,t}$ represents the total bilateral agricultural product trade value between countries *i* and *j*. The modularity index is defined as

(17) $$Q^{t}=\frac{1}{2m^{t}}\sum_{i}\sum_{j}\left[W_{\mathrm{ij}}^{u,t}-\frac{k_{i}^{t}k_{j}^{t}}{2m^{t}}\right]\delta (c_{i},c_{j})$$

where $k_{i}^{t}=\sum_{j}W_{\mathrm{ij}}^{u,t}$ is the weighted degree of country *i*, $m^{t}=\frac{1}{2}\sum_{i}\sum_{j}W_{\mathrm{ij}}^{u,t}$ is the total edge weight of the undirected weighted network, and *δ*(*c_i_*, *c_j_*) equals 1 if countries *i* and *j* belong to the same community and 0 otherwise.

This study uses a greedy modularity optimization algorithm. Each country is initially treated as a separate community. The algorithm then repeatedly merges the pair of communities that produces the largest increase in modularity. The process stops when no further merger can improve *Q^t^*. The resulting communities indicate groups of BRICS members with relatively stronger internal agricultural product trade relations.

### 3.5. QAP Analysis Method

To identify the driving factors of agricultural product trade relations among BRICS members, this study applies the quadratic assignment procedure (QAP) based on the analytical framework of agricultural trade attraction, supply–demand matching and multidimensional trade distance. QAP is suitable for network data because dyadic observations are not independent [43]. In the BRICS agricultural product trade network, trade relations that share the same exporter or importer may be correlated, which violates the independence assumption of conventional correlation and regression methods. QAP addresses this problem through matrix permutation: the rows and corresponding columns of a matrix are simultaneously permuted to generate an empirical reference distribution for statistical inference.

#### 3.5.1. QAP Correlation Analysis Method

QAP correlation analysis is used to examine the bivariate association between the agricultural product trade intensity matrix and each explanatory matrix. For each explanatory variable *X^t^*, the Pearson correlation coefficient between the vectorized off-diagonal elements of *Y^t^* and *X^t^* is first calculated:

(18) $$r^{t}=\mathrm{coor}(\mathrm{vec}(Y_{\mathrm{ij}}^{t}),\mathrm{vec}(X_{\mathrm{ij}}^{t})),i\neq j$$

After obtaining the observed correlation coefficient, the QAP repeatedly permutes the rows and corresponding columns of the explanatory matrix, recalculates the correlation coefficient for each permutation, and compares the observed coefficient with the empirical distribution generated by the permutations. The significance level is obtained as the proportion of permuted coefficients whose absolute values are greater than or equal to the absolute value of the observed coefficient. This study uses 5000 permutations for the QAP correlation analysis.

#### 3.5.2. QAP Regression Analysis Method

QAP regression is used to examine the joint effects of multiple explanatory matrices on agricultural product trade intensity. The regression model is specified as

(19) $${\mathrm{Exp\_agr}}_{\mathrm{ij}}^{t}=\beta_{0}+\beta_{1}{\mathrm{Sup}}_{i}^{t}+\beta_{2}{\mathrm{Pre}}_{j}^{t}+\beta_{3}{\mathrm{Com}}_{\mathrm{ij}}^{t}+\beta_{4}{\mathrm{Dis\_geo}}_{\mathrm{ij}}^{t}+\beta_{5}{\mathrm{Dis\_cul}}_{\mathrm{ij}}^{t}+\beta_{6}{\mathrm{Dis\_ins}}_{\mathrm{ij}}^{t}$$

where ${\mathrm{Exp\_agr}}_{\mathrm{ij}}^{t}$ is the logarithm of agricultural product exports from country *i* to country *j*. ${\mathrm{Sup}}_{i}^{t}$ represents the agricultural supply capacity of the exporting country, ${\mathrm{Pre}}_{j}^{t}$ represents the food-security pressure of the importing country, ${\mathrm{Com}}_{\mathrm{ij}}^{t}$ represents supply–demand complementarity, ${\mathrm{Dis\_geo}}_{\mathrm{ij}}^{t}$ represents geographical distance, ${\mathrm{Dis\_cul}}_{\mathrm{ij}}^{t}$ represents cultural distance, and ${\mathrm{Dis\_ins}}_{\mathrm{ij}}^{t}$ represents institutional distance.

The regression is estimated using the off-diagonal elements of the matrices. To make coefficients comparable across variables measured in different units, all variables are standardized before regression. Statistical significance is assessed using the QAP permutation procedure. A positive coefficient indicates that the corresponding factor is associated with stronger agricultural product trade relations, whereas a negative coefficient indicates weaker trade relations. By combining QAP correlation and QAP regression, this study first identifies pairwise matrix associations and then evaluates the relative explanatory power of multiple factors under network dependence.

#### 3.5.3. Variable Selection

Variable selection is based on the analytical framework developed in this study, which integrates the extended gravity model, the characteristics of agricultural trade and multidimensional trade distance.

1. Dependent Variable

Agricultural trade intensity (Exp_agr). This variable is measured by the logarithm of one plus the export value of agricultural products from country i to country j. Agricultural products are defined according to HS01-HS24. The logarithmic transformation reduces the influence of extreme trade values while preserving differences in trade intensity. This variable forms the dependent matrix in the QAP correlation and regression analyses.

2. Explanatory Variables

Agricultural supply capacity (Sup). The agricultural supply capacity reflects the exporter’s ability to provide agricultural products to other BRICS members. In this study, fertilizer consumption intensity is used to capture the input-based dimension of the agricultural supply capacity. Chemical fertilizer is a key material input in modern agricultural production, and fertilizer consumption per hectare of arable land reflects the intensity of agricultural input use and the degree of production intensification [44]. Compared with indicators such as soil quality, fertilizer consumption is available on a comparable cross-country and cross-year basis and is directly related to land productivity and output potential. Accordingly, Sup is measured by the fertilizer consumption intensity of the exporting country, expressed as the logarithm of one plus fertilizer consumption per hectare of arable land. A higher value indicates a stronger agricultural input intensity and greater potential export supply capacity.

Food-security pressure (Pre). Food-security pressure captures the importer’s potential demand for external agricultural products under resource constraints [45]. In countries with limited arable land per capita, domestic agricultural production may face stronger land-resource pressure, which can increase reliance on agricultural products obtained through trade. Pre is therefore measured by the inverse of arable land per capita in the importing country. A higher value indicates stronger land constraints and a greater potential need to obtain agricultural products through external markets.

Supply–demand complementarity (Com). Supply–demand complementarity reflects the match between the exporter’s food export orientation and the importer’s food import orientation [46]. It is measured by multiplying the standardized food export share of the exporting country by the standardized food import share of the importing country. A higher value indicates stronger potential complementarity between export supply and import demand.

Geographical distance (Dis_geo). Geographical distance captures spatial separation and transport costs between two countries. It is measured by the natural logarithm of the capital-to-capital distance between the exporter and the importer [47]. In the extended gravity framework, a greater distance is generally expected to weaken trade intensity.

Cultural distance (Dis_cul). Cultural distance reflects language-related communication and transaction costs. It is measured as one minus the common-language indicator [48]. A larger value indicates a greater linguistic or cultural distance between two countries.

Institutional distance (Dis_ins). Institutional distance captures differences in governance quality, regulatory systems, rule enforcement and institutional environments between countries [49]. Agricultural trade is sensitive to institutional differences because it involves customs procedures, inspection and quarantine, food safety standards, certification and contract enforcement. This study measures institutional distance using six worldwide governance indicators: voice and accountability, political stability and absence of violence/terrorism, government effectiveness, regulatory quality, rule of law and control of corruption. Following the multidimensional distance-index logic [30], institutional distance is calculated as

(20) $${\mathrm{Dis\_ins}}_{\mathrm{ij}}^{t}=\frac{1}{6}\sum_{c=1}^{6}\frac{{\left({\mathrm{WGI}}_{\mathrm{ic}}^{t}-{\mathrm{WGI}}_{\mathrm{jc}}^{t}\right)}^{2}}{\mathrm{Var}({\mathrm{WGI}}_{c}^{t})}$$

where ${\mathrm{WGI}}_{\mathrm{ic}}^{t}$ and ${\mathrm{WGI}}_{\mathrm{jc}}^{t}$ denote the governance scores of countries *i* and *j* on dimension *c* in year *t*, and $\mathrm{Var}({\mathrm{WGI}}_{c}^{t})$ is the sample variance of governance dimension *c*. A higher value indicates a greater institutional distance.

Data cleaning, country-pair matrix construction, network indicator calculation, and QAP correlation and regression analyses were implemented in Python 3.12 using pandas and NumPy. All variables are organized into country-pair matrices consistent with the directed trade matrix. The above variables and data sources are summarized in Table 1.

## 4. Results

### 4.1. Evolutionary Characteristics of BRICS Agricultural Products Trade Networks

#### 4.1.1. Overall Characteristics of the Trade Network

**Figure 2:**
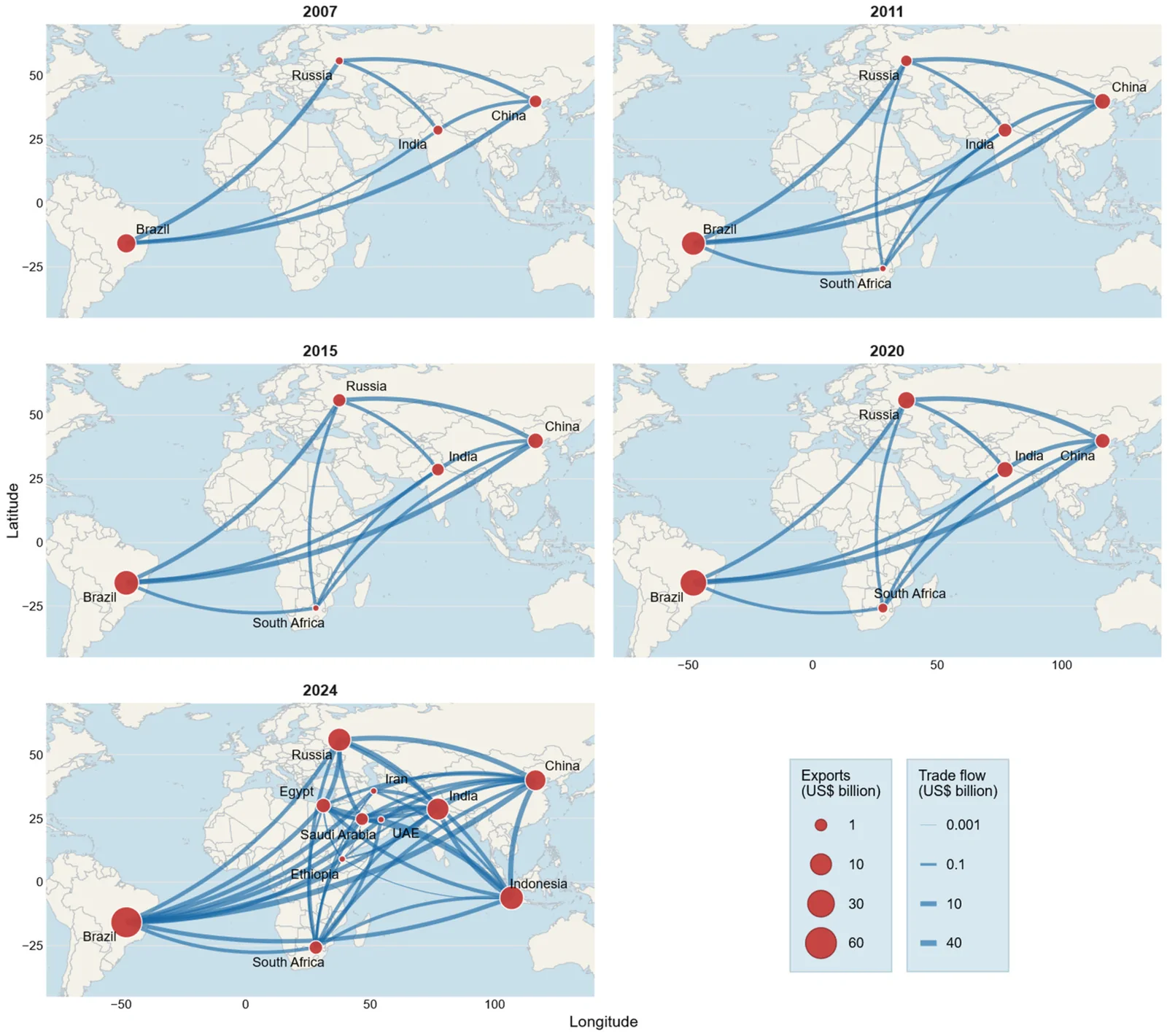
Evolution of the BRICS agricultural product trade network pattern.

Figure 2 presents the spatial structure of agricultural product trade among BRICS countries. Node size indicates each country’s exports to other BRICS members, and line width indicates total trade between each country pair. In 2007, trade ties were mainly concentrated in the Brazil–China, Brazil–Russia and China–Russia pairs, and total intra-network trade was only USD 9.780 billion, indicating a small and highly concentrated network. After South Africa joined in 2011, the network extended to southern Africa. In 2015 and 2020, Brazil–China continued to dominate the network, while Russia–China and India–China trade also expanded. By 2020, the total intra-network trade had increased to USD 42.599 billion. After the 2024 enlargement, the network became more complex and cross-regional, spanning South America, East Asia, South Asia, Southeast Asia, the Middle East and Africa, whose total trade reached USD 125.863 billion. The largest bilateral relations were Brazil–China (USD 43.282 billion), China–Indonesia (USD 11.783 billion), China–Russia (USD 10.266 billion), India–Indonesia (USD 6.009 billion) and Egypt–Russia (USD 4.220 billion). Overall, the network evolved from a small structure dominated by several core bilateral ties into a broader cross-regional network.

Table 2 reports the overall characteristics of the BRICS agricultural product trade network. BRIC-4 refers to the network constructed among Brazil, Russia, India and China; BRICS-5 refers to the network constructed among Brazil, Russia, India, China and South Africa; and BRICS-11 refers to the network constructed among the eleven BRICS countries after the 2024 enlargement. To improve comparability before and after enlargement, this study additionally constructs a 2024 BRICS-5 network using only the original five members as a fixed-membership comparison.

The results show that density, reciprocity, average path length and global efficiency all equaled 1 during 2011, 2015 and 2020 for the original five BRICS members, indicating that the five-member BRICS grouping had formed a fully connected agricultural product trade network before enlargement. The 2024 BRICS-5 network also remained fully connected, whereas in the 2024 BRICS-11 network, density fell to 0.691, reciprocity declined to 0.509, average path length rose to 1.050, and global efficiency dropped to 0.709. This comparison suggests that the expansion of network scale was accompanied by structural sparsification after enlargement. Total agricultural product trade increased markedly, but reciprocal trade linkages among new members and between some new and original members had not yet been fully established.

#### 4.1.2. Node-Level Characteristics of the Trade Network

Node-level indicators are used to examine the structural positions of member states within the agricultural product trade network. The network remained fully connected from 2007 to 2020; therefore, the in-closeness and out-closeness centralities of all member states equaled 1, while betweenness centrality was 0. The node-level characteristics of the enlarged BRICS network in 2024 are reported in Table 3.

As shown in Table 3, in-closeness centrality values were relatively similar across most members in 2024, mainly around 0.700. Ethiopia and the United Arab Emirates had slightly higher values of 0.711, whereas Iran had the lowest value of 0.640. Out-closeness centrality showed greater differentiation. Brazil, China, Egypt, India, Indonesia and South Africa all had values of 1.000, Saudi Arabia and Russia had lower values of 0.909 and 0.769, respectively, and Ethiopia, Iran and the United Arab Emirates had values of 0. Betweenness centrality was generally low: Brazil, China, Egypt, India, Indonesia and South Africa had values of 0.007, Saudi Arabia had a value of 0.003, and Ethiopia, Iran, Russia and the United Arab Emirates had values of 0. Overall, the 2024 network was mainly connected through direct trade relations, with limited intermediary roles among members.

**Figure 3:**
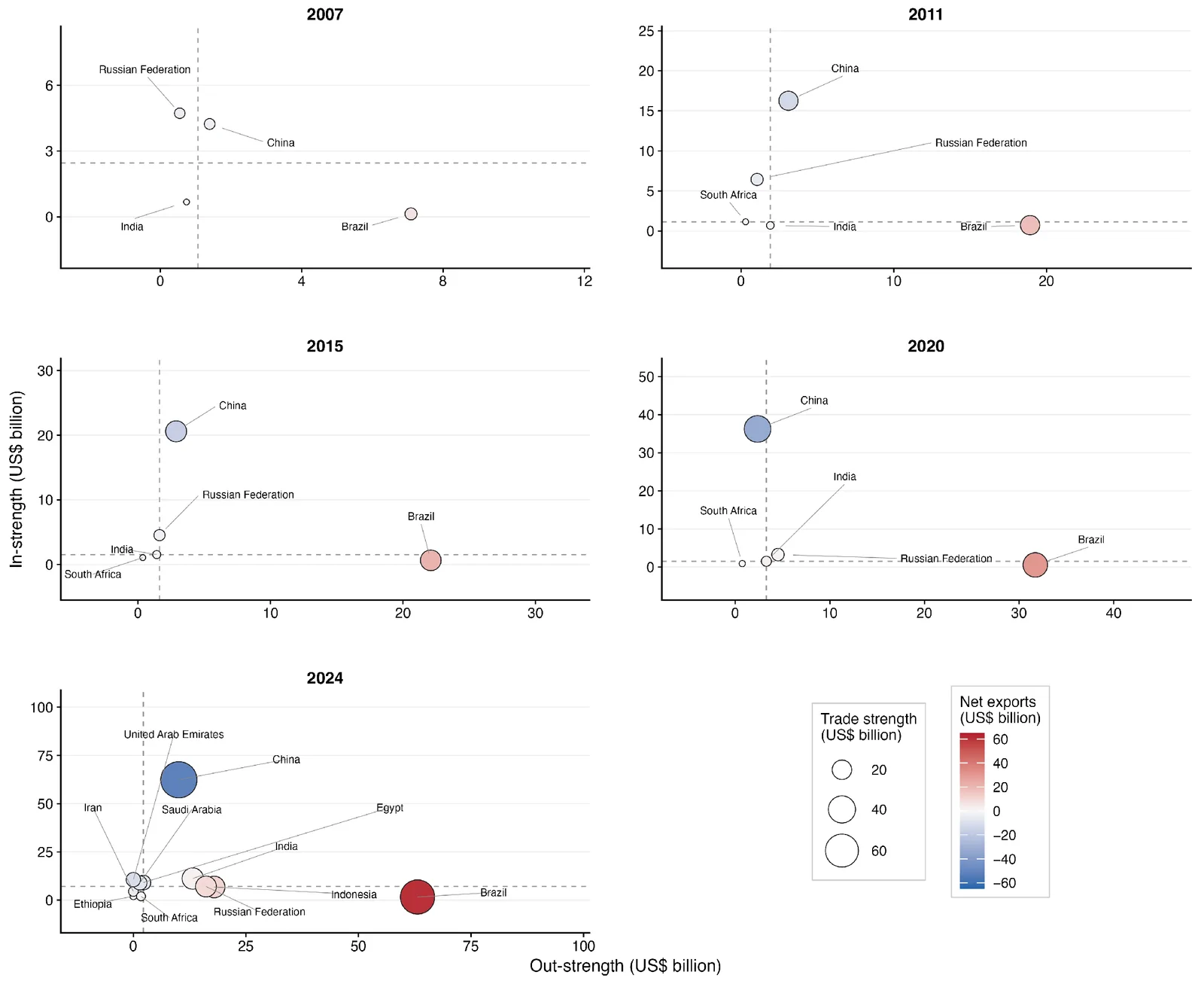
Out-strength and in-strength role distribution of nodes in BRICS agricultural product trade networks.

Figure 3 illustrates the in-strength, out-strength and net export structure of BRICS member states in agricultural trade. Brazil and China consistently occupied the high-export and high-import positions, respectively, from 2007 to 2020, while India, Russia and South Africa showed more fluctuating patterns. After the 2024 enlargement, the differentiation of node roles became more pronounced. Brazil recorded an out-strength of approximately USD 63.116 billion and a net export value of around USD 61.516 billion, remaining the most prominent export-dominant node. China recorded an in-strength of about USD 62.358 billion and a net import value of roughly USD 52.256 billion, functioning as the core import-dominant node. Russia had an out-strength of USD 16.133 billion, an in-strength of USD 7.029 billion and a net export value of USD 9.104 billion. India recorded an out-strength of USD 13.198 billion and an in-strength of USD 11.163 billion in 2024, indicating a relatively balanced role with a small net export scale.

Overall, Brazil remains in the high out-strength and low in-strength zone, while China remains in the high in-strength zone. Russia and Indonesia moved into a relatively high out-strength zone after enlargement, whereas the United Arab Emirates, Saudi Arabia and Egypt were closer to the import-demand side. This pattern indicates that the enlarged BRICS agricultural product trade network has not produced homogeneous inter-member relations. Instead, a hierarchical division of labor has emerged among supply hubs, demand hubs, balanced nodes and low-participation nodes.

#### 4.1.3. Community Characteristics of Trade Networks

Community structure further reveals internal grouping relations within the BRICS agricultural product trade network. As shown in Table 4, the clustering coefficients of the BRICS agricultural product trade network remained above 0.9 from 2007 to 2024, indicating that countries connected through agricultural product trade tended to form closely linked local neighborhoods. Modularity further reflects the degree of structural differentiation among communities. The modularity values remained very low before enlargement, rising from 0.0070 in 2011 to 0.0165 in 2020, which suggests that community boundaries were weak during the original-member period. In 2024, modularity increased to 0.1191, indicating that subgroup differentiation became more visible after enlargement.

In terms of the number of communities, the agricultural product trade network of the original four BRICS countries formed only one community in 2007. In 2011 and 2015, the network was divided into two communities: Brazil, China and Russia formed one core community, while India and South Africa formed the other. In 2020, Brazil and China were grouped into one community, while India, Russia and South Africa formed the other, indicating a shift in the linkage pattern between demand and supply nodes. After the 2024 enlargement, the network still consisted of two communities: the first included Brazil, China, Ethiopia and Iran, and the second included Egypt, India, Indonesia, Russia, Saudi Arabia, South Africa and the United Arab Emirates. Modularity rose from 0.0165 in 2020 to 0.1191 in 2024. This indicates that group structures became clearer after enlargement, although overall modularity remained low. The BRICS agricultural product trade network therefore showed some community differentiation but did not split into closed regional blocs.

### 4.2. Driving Mechanisms of Trade Network Patterns

#### 4.2.1. QAP Correlation Analysis

QAP correlation analysis is used to examine bivariate relationships between the explanatory-factor matrices and the agricultural product trade intensity matrix. As shown in Table 5, the overall correlations between most variables and trade intensity were weak before enlargement. Specifically, agricultural supply capacity showed a weakly significant positive correlation in 2011, with a correlation coefficient of 0.627.

The correlation results for 2024 show that geographical distance had a significant positive correlation with trade intensity, while institutional distance had a significant negative correlation. The positive coefficient for geographical distance should not be interpreted as evidence that distance promotes trade. Rather, it reflects the cross-distance nature of BRICS agricultural product trade linkages: major agricultural product trade flows occur among members located far apart, so geographical distance does not operate as a stable constraint in the same way as it often does in geographically proximate regional trade networks. By contrast, the negative correlation for institutional distance is consistent with the extended gravity-model expectation that institutional differences increase trade costs. Larger differences in governance systems, rule enforcement and regulatory capacity may raise the costs of coordination, compliance and expectation alignment, thereby weakening trade intensity. Supply–demand complementarity and cultural distance did not show stable significant correlations across the five benchmark years, suggesting that BRICS agricultural product trade cooperation is not primarily grounded in shared language, historical–cultural proximity or regional integration.

#### 4.2.2. QAP Regression Analysis

**Figure 4:**
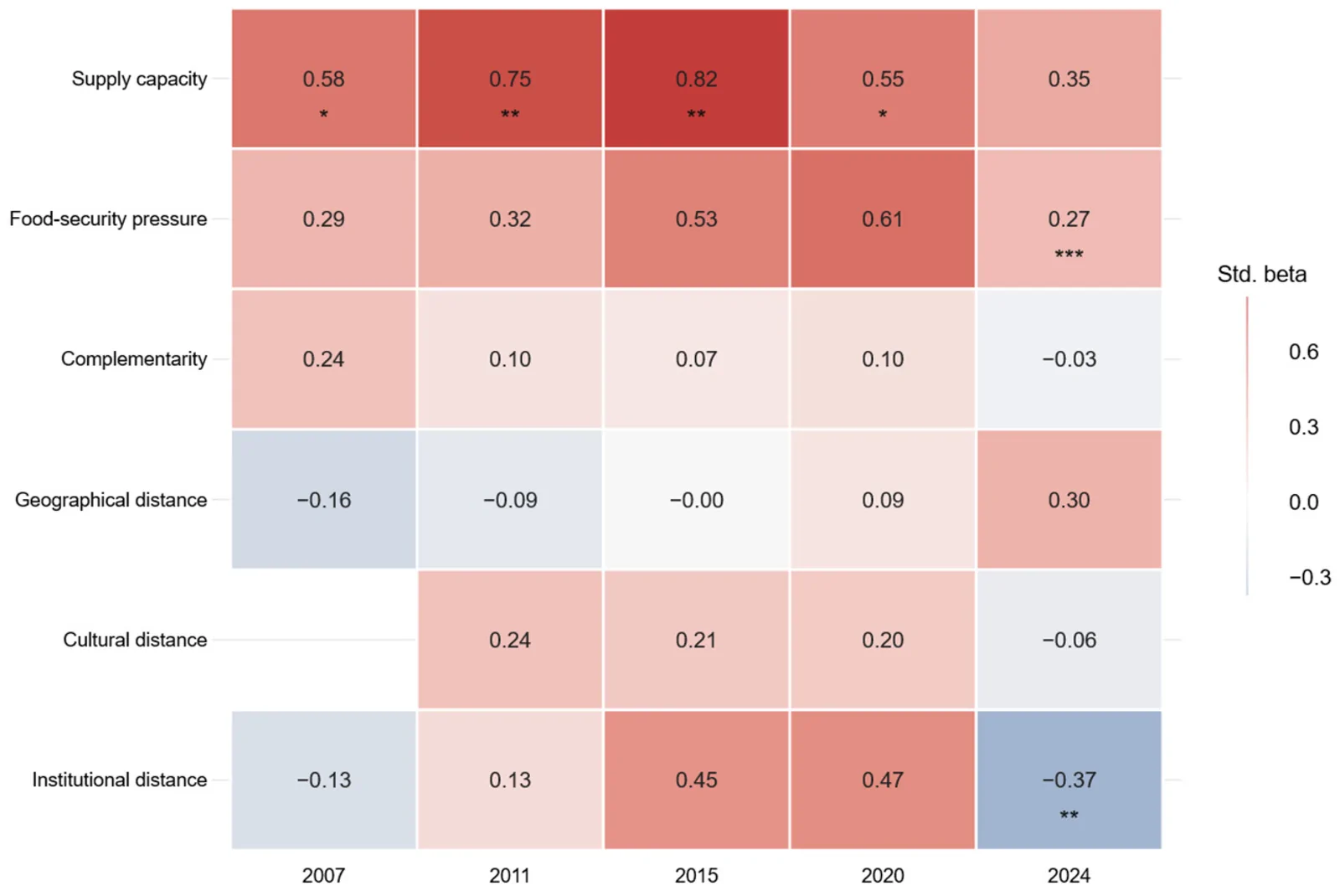
Heatmap of QAP regression coefficients for BRICS agricultural product trade networks. Note: *** *p* < 0.01, ** *p* < 0.05, * *p* < 0.10.

QAP regression analysis further examines the influencing factors of bilateral agricultural product trade intensity within the BRICS network from the perspectives of trade attraction, trade matching and trade resistance. As shown in Figure 4, exporters’ agricultural supply capacity had positive coefficients in 2007, 2011, 2015 and 2020. The coefficients were significant at the 5% level in 2011 and 2015 and at the 10% level in 2020. This indicates that, before enlargement, exporters’ agricultural input intensity and supply capacity played an important role in shaping intra-BRICS agricultural product export intensity. In 2024, the coefficient of agricultural supply capacity remained positive but became statistically insignificant, suggesting that the explanatory role of exporter supply capacity weakened in the enlarged network. At the same time, import demand pressure and institutional distance became more prominent factors in explaining bilateral trade intensity.

For import demand, the 2024 QAP regression produced a significantly positive coefficient of food-security pressure, with a standardized coefficient of 0.271 at the 1% level. This result is consistent with the entry of members such as Saudi Arabia, the United Arab Emirates and Egypt, which face land or water-resource constraints and have strong agricultural import demands. Import demand pressure therefore became an important factor in explaining trade intensity in 2024. From this perspective, the expansion of the BRICS agricultural product trade network reflects both membership enlargement and the adjustment of supply–demand relations under resource constraints.

Institutional distance was significantly negative in the 2024 QAP regression at the 5% level. After agricultural supply capacity, import demand pressure, supply–demand complementarity, geographical distance and cultural distance were included in the model, institutional distance still showed a negative relationship with agricultural product trade intensity among BRICS members. Agricultural product trade involves inspection and quarantine, quality standards, customs clearance and food safety regulation. Differences in governance environments may increase the costs of obtaining regulatory information, adapting to administrative requirements and ensuring compliance, potentially constraining bilateral trade. For the enlarged BRICS grouping, members span multiple regions and differ substantially in governance systems and regulatory capacity, making institutional coordination an important condition for deepening trade connectivity.

Geographical distance and cultural distance did not show stable significance in the QAP regressions, which was consistent with the QAP correlation results. This finding suggests that BRICS agricultural product trade is organized through cross-regional cooperation among emerging market and developing economies. Large trade flows such as Brazil–China, Russia–China and Indonesia–China show that high-value agricultural product trade linkages can extend across long distances. In this network, supply–demand relations, resource endowments and institutional coordination had stronger explanatory relevance than geographical or cultural proximity.

To examine the robustness of the QAP regression results, this study further conducted robustness checks using alternative indicators. First, the original agricultural supply capacity variable was replaced by an agricultural supply capacity index. This index captures six dimensions, including agricultural input intensity, resource base, production performance, output scale, production efficiency and external supply capacity. It was constructed using the entropy method based on fertilizer consumption per hectare of arable land, arable land area, crop production index, cereal production, cereal yield and global agricultural export scale. The corresponding QAP regression results are reported in Appendix A, Table A2. Second, the original import demand pressure variable was replaced by water stress. Water stress is measured by the level of water stress, defined as freshwater withdrawal as a proportion of available freshwater resources, and is assigned according to the importing country to construct a directed country-pair matrix. The corresponding QAP regression results are reported in Appendix A, Table A3. The robustness results are broadly consistent with the baseline QAP regressions.

Taken together, exporters’ agricultural supply capacity had stronger explanatory power for intra-BRICS trade intensity before enlargement. After enlargement, import demand pressure and institutional distance became more prominent explanatory factors. This shift indicates that the evolution of the BRICS agricultural product trade network includes both membership expansion and the adjustment of supply–demand relations within a broader cross-regional agri-food system. From a food-security-related perspective, the entry of members with strong import demand strengthens the network’s role as a trade channel between resource-abundant and resource-constrained economies. At the same time, the negative coefficient of institutional distance indicates that trade connectivity is linked to the compatibility of customs procedures, food safety standards, inspection and quarantine systems and regulatory enforcement. Improving the BRICS agricultural product trade network therefore requires attention to supply-side nodes such as Brazil, Russia and Indonesia, demand-side members such as China and several Middle Eastern and African economies, and institutional coordination that can reduce cross-regional transaction costs.

## 5. Discussion and Policy Implications

### 5.1. Discussion

The enlargement of the BRICS agricultural product trade network did not displace its main hubs. Trade links extended to more members, while the two communities remained connected by substantial cross-community trade. The coexistence of expanding links and persistent hubs is consistent with patterns identified in global agricultural trade networks [3,25]. China–ASEAN and RCEP studies also identify central countries within their regional networks [28,29]. The BRICS results also add a cross-regional case: geographical distance was not consistently associated with trade links across model specifications, whereas geographical accessibility played an important role in the China–ASEAN network [28].

The QAP results further identify factors associated with BRICS trade links. Food-security pressure was positively associated with trade, while institutional distance showed a negative association in 2024. The result is consistent with research on China’s trade with Belt and Road countries [49] and with evidence that agri-food trade relationships last longer between partners with more similar institutional quality [50]. By contrast, aggregate trade complementarity showed a weak association. Research on Belt and Road agricultural trade finds that competition and complementarity vary across country pairs [42], suggesting that a network-level measure may not capture every bilateral relationship. These comparisons help interpret the BRICS agricultural product trade network results.

### 5.2. Policy Implications

Taken together, the empirical results and discussion show that the enlarged BRICS agricultural product trade network reached more members in 2024 but had a lower density, reciprocity and global efficiency than the five-member network. And institutional distance was negatively associated with trade links. Accordingly, policy efforts should focus on improving network connectivity, coordinating supply–demand roles, reducing institutional coordination costs and supporting cross-regional trade channels.

First, BRICS agricultural cooperation should move from a primary emphasis on trade-volume expansion toward stronger network connectivity and reciprocity. The empirical results show that the 2024 enlargement substantially increased the scale of the agricultural product trade network, while density, reciprocity and global efficiency declined in the enlarged network. This suggests that some new members had not yet developed stable two-way trade links with original members or with other new members. Future cooperation should therefore place greater emphasis on trade linkages between new and original members and among new members. BRICS could use the agriculture ministers’ meeting and existing economic and trade cooperation mechanisms to build information-sharing systems for key agricultural products, organize product-specific supply–demand matching and formulate practical bilateral cooperation lists. These measures would help low-participation nodes establish more stable reciprocal trade links with core supply and demand nodes.

Second, BRICS members should improve supply–demand coordination according to their differentiated roles in agricultural product trade. The results show that Brazil has long maintained an export-dominant position, while China has remained import-dominant. Russia and Indonesia have strengthened their supply-side roles after enlargement, whereas Egypt, Saudi Arabia and the United Arab Emirates are closer to the import-demand side. This structural differentiation suggests that cooperation should recognize differences in resource endowments, production capacity and demand structures among members. Stabilizing the export capacity of Brazil, Russia and Indonesia, while responding to the import demand of China and Middle Eastern and African members, would help form a more stable supply–demand coordination mechanism and reduce excessive concentration in a limited number of trade corridors.

Third, BRICS should reduce coordination costs associated with institutional distance and strengthen alignment of agricultural product trade rules. The QAP results show that institutional distance had a significantly negative coefficient in 2024, suggesting that differences in governance systems, the regulatory capacity and rule enforcement were closely related to a weaker trade intensity. Agricultural product trade involves food safety, inspection and quarantine, quality certification, customs supervision and transport timeliness, all of which require institutional coordination. BRICS members could prioritize cooperation in inspection and quarantine for key agricultural products, mutual recognition of standards, electronic certificates and customs-clearance facilitation. For country pairs with close trade potential but large institutional gaps, pilot programs for agricultural product trade facilitation could be used to improve trade connectivity through institutional coordination.

Fourth, BRICS should make better use of its cross-regional cooperation framework to support long-distance agricultural product trade. The empirical results show that geographical and cultural distance did not exert stable inhibitory effects, reflecting the cross-regional nature of BRICS agricultural product trade linkages. Future cooperation should not rely on geographical proximity as the main basis for prioritizing agricultural product trade partnerships. Cooperation priorities can be organized around supply capacity, import demand and institutional coordination conditions. Improvements in cross-regional logistics corridors, cold-chain transport, trade finance and digital trade platforms would help strengthen the stability and accessibility of long-distance agricultural product trade and support the transition from scale expansion toward deeper network integration.

## 6. Conclusions, Limitations and Future Research

### 6.1. Conclusions

Using BRICS agricultural product trade data for 2007, 2011, 2015, 2020 and 2024, this study constructs directed weighted trade networks and applies QAP correlation and regression analyses to answer how the BRICS agricultural product trade network has evolved, and which factors are associated with its structural changes. The main findings are as follows:

First, the BRICS agricultural product trade network continued to expand in scale, while the 2024 enlargement brought both total-volume growth and structural sparsification. From 2007 to 2020, total agricultural product trade among BRICS members increased from USD 9.780 billion to USD 42.599 billion. After the 2024 enlargement, it further rose to USD 125.863 billion, about 2.95 times the 2020 level. Enlargement broadened the spatial coverage of the network, extending trade linkages across South America, East Asia, South Asia, the Middle East, Africa and Southeast Asia. However, network density fell to 0.691, reciprocity declined to 0.509 and global efficiency decreased to 0.709 in 2024. This indicates that new members increased overall trade volume, but reciprocal trade ties among new members and between some new and original members remained insufficiently developed.

Second, node roles in the BRICS agricultural product trade network became increasingly differentiated. From 2007 to 2020, Brazil–China, Brazil–Russia and China–Russia constituted core bilateral trade relationships. After the 2024 enlargement, major bilateral trade connections included Brazil–China, Indonesia–China, Russia–China, Russia–Egypt and Indonesia–India. Node-strength results show that Brazil recorded a net export value of approximately USD 61.516 billion in 2024, making it the most prominent export-dominant node, while China recorded a net import value of around USD 52.256 billion, functioning as the leading import-dominant node. Russia and Indonesia performed strong supply-side functions, whereas the United Arab Emirates, Saudi Arabia and Egypt were closer to the import-demand side. The enlarged network therefore formed a hierarchical division of labor among supply hubs, demand hubs, balanced nodes and low-participation nodes.

Third, the BRICS agricultural product trade network showed strong agglomeration but no closed community segmentation. The clustering coefficient remained above 0.9 in all benchmark years, indicating strong local clustering among members. The network formed one community in 2007 and two communities in 2011, 2015 and 2020, with low modularity values. After enlargement, modularity increased from 0.0165 in 2020 to 0.1191, suggesting that group structures became more visible. However, modularity remained relatively low overall. The network therefore displayed community differentiation without being divided into closed regional blocs, and cross-community trade linkages remained important. This pattern is consistent with the cross-regional nature of BRICS cooperation and suggests that agricultural product trade can connect supply and demand beyond geographically proximate blocs, which is relevant for improving the flexibility of agri-food supply systems.

Fourth, the driving factors of the BRICS agricultural product trade network varied across stages. QAP correlation analysis shows that correlations between most variables and trade intensity were weak before enlargement, with agricultural supply capacity showing a weakly significant positive correlation only in 2011. This may be related to the high connectivity of the pre-enlargement network, in which trade differences were reflected mainly in trade volume rather than in the presence or absence of trade ties. In 2024, institutional distance was significantly negatively correlated with trade intensity, indicating that differences in governance systems, rule enforcement and regulatory capacity increased coordination and compliance costs in agricultural product trade.

Fifth, trade intensity was mainly associated with exporters’ agricultural supply capacity before enlargement, whereas food-security pressure and institutional distance became more prominent after enlargement. The QAP regression results show that exporters’ agricultural supply capacity was generally positive during 2007–2020 and reached significance in 2011, 2015 and 2020. In 2024, food-security pressure was significantly positive, institutional distance was significantly negative, and exporters’ supply capacity was no longer statistically significant. This change reflects the entry of new members with strong import demand, including Saudi Arabia, the United Arab Emirates and Egypt. It also suggests that wider institutional differences among enlarged BRICS members have become an important constraint on deeper network integration. For agricultural product trade, such institutional differences may affect food safety regulation, inspection and quarantine, certification and customs clearance, thereby influencing the stability and accessibility of cross-border agricultural supply. The unstable significance of geographical and cultural distance further indicates that BRICS agricultural product trade is not a typical geographically proximate regional trade network, but a cross-regional institutional trade network.

### 6.2. Limitations and Future Research

This study still has several limitations. First, the time window after BRICS enlargement is relatively short. The empirical analysis uses five benchmark years, namely 2007, 2011, 2015, 2020 and 2024. Since 2024 is the first year after the recent enlargement, the trade relations of new members may still be in an early adjustment stage. The current results therefore mainly reflect the initial network structure after enlargement, while the lagged and long-term effects of enlargement require further observation. Second, this study uses aggregate HS01-HS24 agricultural product trade values and does not distinguish specific product categories. Agricultural products include cereals, soybeans, meat, fruits, vegetables, beverages, processed foods and other commodities. These products differ substantially in production conditions, trade patterns, nutritional functions, resource use and supply risks. Therefore, the aggregate network can reveal the overall structure of BRICS agricultural product trade, but it cannot fully identify product-specific trade relations or the deeper commodity-level structure of the network. Third, the method used in this study has limitations in explaining external shocks. Social network analysis and QAP are useful for describing network structure and examining matrix-based relationships, but they do not directly identify the effects of external shocks. Events such as the COVID−19 pandemic, the Russia–Ukraine conflict, Black Sea trade disruptions, sanctions, international price fluctuations and short-term market substitution may have affected the 2020 and 2024 trade patterns. These factors are not explicitly incorporated into the current model.

Future research can be extended in several directions. First, continuous annual data can be used to examine the lagged effects and long-term evolution of BRICS enlargement. This would help distinguish temporary adjustment from stable structural change in the agricultural product trade network. Second, product-specific networks can be constructed for cereals, soybeans, meat, fruits, vegetables and processed foods to compare the evolution patterns of different agricultural products and clarify the deeper commodity-level structure of BRICS agricultural product trade. Third, future studies can adopt dynamic network models, multilayer network models or other causal identification strategies to incorporate external shocks and examine how they reshape trade relations. Fourth, trade value data can be combined with trade quantity, calories, protein, nutrition, virtual water and carbon-footprint data to assess the implications of agricultural product trade for food security, nutrition and sustainable development more directly.

## Figures and Tables

**Table 1:** Variable selection and data sources.

Variable	Measurement	Data Sources
Exp_agr	*Ln*(1 + *Export*_*ij*)	UN Comtrade
Sup	*Ln*(1 + *Input*_*Fer*_*i*)	FAOSTAT
Pre	1/*AraLand*_*pc*_*j*	FAOSTAT
Com	*Z*(*FoodE*_*i*) × *Z*(*FoodI*_*j*)	FAOSTAT
Dis_geo	*Ln*(*Geo*_*ij*)	CEPII
Dis_cul	1 − *ComLan*_*ij*	CEPII
Dis_ins	$\frac{1}{6}\sum_{c=1}^{6}\frac{{\left(\mathrm{WG}I_{\mathrm{ic}}-\mathrm{WG}I_{\mathrm{jc}}\right)}^{2}}{\mathrm{Var}(\mathrm{WG}I_{c})}$	World Bank

**Table 2:** Overall characteristics of BRICS agricultural product trade networks.

Year	Network	Node	Edge	Density	Reciprocity	Average Path Length	Global Efficiency
2007	BRIC-4	4	12	1.000	1.000	1.000	1.000
2011	BRICS-5	5	20	1.000	1.000	1.000	1.000
2015	BRICS-5	5	20	1.000	1.000	1.000	1.000
2020	BRICS-5	5	20	1.000	1.000	1.000	1.000
2024	BRICS-5	5	20	1.000	1.000	1.000	1.000
2024	BRICS-11	11	76	0.691	0.509	1.050	0.709

**Table 3:** Node-level characteristics of BRICS agricultural product trade networks in 2024.

Year	Country	In-Closeness Centrality	Out-Closeness Centrality	Betweenness Centrality
2024	Brazil	0.700	1.000	0.007
2024	China	0.700	1.000	0.007
2024	Egypt	0.700	1.000	0.007
2024	Ethiopia	0.711	0.000	0.000
2024	India	0.700	1.000	0.007
2024	Indonesia	0.700	1.000	0.007
2024	Iran	0.640	0.000	0.000
2024	Russian Federation	0.700	0.769	0.000
2024	Saudi Arabia	0.700	0.909	0.003
2024	South Africa	0.700	1.000	0.007
2024	United Arab Emirates	0.711	0.000	0.000

**Table 4:** Community characteristics of BRICS agricultural product trade networks.

Year	Clustering Coefficient	Modularity	Communities
2007	1.000	−0.0000	1
2011	1.000	0.0070	2
2015	1.000	0.0067	2
2020	1.000	0.0165	2
2024	0.908	0.1191	2

**Table 5:** QAP correlation analysis of BRICS agricultural product trade networks.

Variable	2007	2011	2015	2020	2024
Sup	0.446	0.627 *	0.512	0.283	0.414
Pre	0.144	0.121	0.223	0.453	0.045
Com	0.043	−0.010	−0.069	−0.063	0.051
Dis_geo	−0.096	0.025	0.058	−0.003	0.393 **
Dis_cul	/	0.238	0.232	0.247	−0.092
Dis_ins	−0.245	−0.230	−0.034	0.218	−0.411 ***

Note: *** *p* < 0.01, ** *p* < 0.05, * *p* < 0.10.

## Data Availability

The original contributions presented in the study are included in the article; further inquiries can be directed to the corresponding author.

## Appendix A

**Table A1:** Products included in the agricultural trade aggregate.

HS Code	WCO Harmonized System Chapter Title
HS01	Live animals.
HS02	Meat and edible meat offal.
HS03	Fish and crustaceans, mollusks and other aquatic invertebrates.
HS04	Dairy produce; birds’ eggs; natural honey; edible products of animal origin, not elsewhere specified or included.
HS05	Products of animal origin, not elsewhere specified or included.
HS06	Live trees and other plants; bulbs, roots and the like; cut flowers and ornamental foliage.
HS07	Edible vegetables and certain roots and tubers.
HS08	Edible fruit and nuts; peel of citrus fruit or melons.
HS09	Coffee, tea, maté and spices.
HS10	Cereals.
HS11	Products of the milling industry; malt; starches; inulin; wheat gluten.
HS12	Oil seeds and oleaginous fruits; miscellaneous grains, seeds and fruit; industrial or medicinal plants; straw and fodder.
HS13	Lac; gums, resins and other vegetable saps and extracts.
HS14	Vegetable plaiting materials; vegetable products not elsewhere specified or included.
HS15	Animal, vegetable or microbial fats and oils and their cleavage products; prepared edible fats; animal or vegetable waxes.
HS16	Preparations of meat, of fish, of crustaceans, mollusks or other aquatic invertebrates, or of insects.
HS17	Sugars and sugar confectionery.
HS18	Cocoa and cocoa preparations.
HS19	Preparations of cereals, flour, starch or milk; pastrycooks’ products.
HS20	Preparations of vegetables, fruit, nuts or other parts of plants.
HS21	Miscellaneous edible preparations.
HS22	Beverages, spirits and vinegar.
HS23	Residues and waste from the food industries; prepared animal fodder.
HS24	Tobacco and manufactured tobacco substitutes; products, whether or not containing nicotine, intended for inhalation without combustion; other nicotine containing products intended for the intake of nicotine into the human body.

**Table A2:** Robustness check replacing agricultural supply capacity.

Variable	2007	2011	2015	2020	2024
Sup	0.653 **	0.806 **	0.911 **	0.657 **	0.405
Pre	0.282	0.292	0.499	0.596	0.229 **
Com	0.207	0.047	0.024	0.081	−0.015
Dis_geo	−0.244	−0.137	−0.036	0.074	0.227
Dis_cul	/	0.148	0.078	0.101	−0.131
Dis_ins	−0.072	0.154	0.487	0.496	−0.351 **

Note: ** *p* < 0.05.

**Table A3:** Robustness check replacing food-security pressure.

Variable	2007	2011	2015	2020	2024
Sup	0.511	0.656 *	0.632 *	0.415	0.339
Pre	0.302	0.149	0.2s88	0.252	0.322 ***
Com	0.249	0.079	0.029	0.053	−0.035
Dis_geo	−0.097	−0.045	0.020	0.057	0.344 *
Dis_cul	/	0.270	0.297	0.274	−0.060
Dis_ins	−0.233	0.022	0.250	0.361	−0.377 **

Note: *** *p* < 0.01, ** *p* < 0.05, * *p* < 0.10.
